# Supplementary material for: Distinct mechanisms mediate speed-accuracy adjustments in cortico-subthalamic networks
Source: eLife. 2017 Jan 31;6:e21481. doi: 10.7554/eLife.21481 (PMC5287713; doi:10.7554/eLife.21481)
Supplement: Supplementary file 1. — Age and disease duration are given in years. UPDRS-III: Unified Parkinson’s disease rating scale part III. DOI: http://dx.doi.org/10.7554/eLife.21481.013 [file elife-21481-supp1.docx]

| Patient | Age | UPDRS-III OFF/ON levodopa | Disease duration | First symptom | Reason for surgery | Medication  (mg / day) | DBS lead | Surgical Centre |
| --- | --- | --- | --- | --- | --- | --- | --- | --- |
| 1 | 62 | 54/31 | 9 | Stiffness | Dyskinesia | Levodopa 400  Amantadine 300  Selgiline 10 | Boston Scientific  DB-2201^TM^ | Oxford |
| 2 | 48 | 46/18 | 6 | Tremor | Tremor | Levodopa 800 | Boston Scientific  DB-2202^TM^ | Oxford |
| 3 | 54 | 61/32 | 8 | Tremor | Motor fluctuations | Levodopa 350  Pramipexole 1.05 | Boston Scientific  DB-2201^TM^ | Oxford |
| 4 | 55 | 27/8 | 6 | Shuffling gate | Dyskinesia | Levodopa 500  Entacapone 1000  Pramipexole 3.15  Rasagiline 1 | Medtronic 3389^TM^ | Oxford |
| 5 | 56 | 17/9 | 3 | Tremor | Dyskinesia | Levodopa 150  Rasagiline 1 | Boston Scientific  DB-2201^TM^ | London |
| 6 | 67 | 32/8 | 8 | Stiffness | Motor fluctuations | Levodopa 250  Entacapone 1400  Rasagiline 1  Ropinirole 12 | Medtronic 3389^TM^ | London |
| 7 | 75 | 31/10 | 11 | Tremor | Gait | Levodopa 150  Pramipexole 3.15  Rasagiline 1 | Medtronic 3389^TM^ | London |
| 8 | 53 | 49/9 | 13 | Stiffness | Gait | Levodopa 600  Selegiline 5  Pramipexole 2.1  Amantadine 100 | Medtronic 3389^TM^ | London |
| 9 | 31 | 54/20 | 5 | Stiffness | Dyskinesia | Levodopa 300  Amantadine 100  Rotigotine 6 | Medtronic 3389^TM^ | London |
| 10 | 68 | 33/15 | 10 | Change in handwriting | Motor fluctuations | Levodopa 650  Entacapone 1000  Rasagiline 1  Ropinirole 8 | Boston Scientific  DB-2202^TM^ | Oxford |
| 11 | 55 | 84/25 | 7 | Stiffness, Tremor | Motor fluctuations, Gait | Levodopa 650  Entacapone 1200  Rotigotine 16 | Boston Scientific  DB-2202^TM^ | Oxford |
